# Highly specific vaginal microbiome signature for gynecological cancers

**DOI:** 10.1515/biol-2022-0850

**Published:** 2024-04-16

**Authors:** Mengzhen Han, Na Wang, Wenjie Han, Xiaolin Liu, Tao Sun, Junnan Xu

**Affiliations:** Department of Breast Medicine 1, Cancer Hospital of China Medical University, Liaoning Cancer Hospital, Shenyang 110000, China; Liaoning Microhealth Biotechnology Co., Ltd, Shanlin Road, Dadong District, Shenyang 110000, China; Department of Breast Medicine 1, Cancer Hospital of Dalian University of Technology, Liaoning Cancer Hospital and Institute, No. 44 Xiaoheyan Road, Dadong District, Shenyang, Liaoning 110000, China

**Keywords:** gynecological cancer, 16S rRNA-seq, microbiome, vagina, random forest

## Abstract

To investigate the vaginal microbiota signature of patients with gynecologic cancer and evaluate its diagnostic biomarker potential. We incorporated vaginal 16S rRNA-seq data from 529 women and utilized VSEARCH to analyze the raw data. α-Diversity was evaluated utilizing the Chao1, Shannon, and Simpson indices, and β-diversity was evaluated through principal component analysis using Bray-Curtis distances. Linear discriminant analysis effect size (LEfSe) was utilized to determine species differences between groups. A bacterial co-abundance network was constructed utilizing Spearman correlation analysis. A random forest model of gynecologic tumor risk based on genus was constructed and validated to test its diagnostic efficacy. In gynecologic cancer patients, vaginal α-diversity was significantly greater than in controls, and vaginal β-diversity was significantly separated from that of controls; there was no correlation between these characteristics and menopause status among the subject women. Women diagnosed with gynecological cancer exhibited a reduction in the abundance of vaginal *Firmicutes* and *Lactobacillus*, while an increase was observed in the proportions of *Bacteroidetes*, *Proteobacteria*, *Prevotella*, *Streptococcus*, and *Anaerococcus*. A random forest model constructed based on 56 genus achieved high accuracy (area under the curve = 84.96%) in gynecological cancer risk prediction. Furthermore, there were discrepancies observed in the community complexity of co-abundance networks between gynecologic cancer patients and the control group. Our study provides evidence that women with gynecologic cancer have a unique vaginal flora structure and microorganisms may be involved in the gynecologic carcinogenesis process. A gynecological cancer risk prediction model based on characteristic genera has good diagnostic value.

## 1 Introduction

Gynecological cancers are a serious threat to women’s health and have a high incidence and mortality rate worldwide [1]. Gynecologic malignancies manifest in the female reproductive organs, with cervical cancer, ovarian cancer, and endometrial cancer being the most prevalent. With the development of next-generation sequencing technologies, the vaginal microbiome, as a uniquely female flora, is increasingly recognized as being able to influence gynecological carcinogenesis [2–5].

Microorganisms are associated with 20% of human malignancies [6]. The role of microorganisms in cancer development is complex, as both cancer-promoting and anticancer functions are attributed to this. The local microbiota forms an important part of the tumor microenvironment. Microbiome alterations causing dysbiosis resulting in impaired barrier function, triggering inflammatory responses, leading to immune dysregulation, and metabolic disturbances may directly or indirectly modulate carcinogenesis, progression, and therapeutic efficacy [7]. The notion that infection and inflammation are risk factors for gynecologic malignancies [8–10] dates back to the 1990s. The preponderance of the colonizing environment in the vaginal region confers vaginal flora a more pronounced influence on female reproductive organs in comparison to other microbiota. The unorthodox composition of vaginal microbiota has the potential to induce carcinogenic changes at the local immunopathological level [11]. Lactobacillus is the hallmark microorganism in vaginal flora studies, and its predominance consistently indicates the health of the female reproductive system [12]. Lactobacillus maintains a healthy female reproductive system and a low vaginal pH by manufacturing lactic acid in defense against pathogens. The dysbiosis of the vaginal microbiota and the migration of invading pathogens along the genital tract may likewise induce pathological processes [13], and this process brings the pathogen and the resulting pathological response closer to the reproductive organs, further acting as a pro-cancer factor. Previous research has shown that microorganisms can enter the uterus through the cervix [14,15]. Furthermore, a noteworthy correlation has been identified between the microbiomes of the fallopian tubes, ovaries, vagina, and cervix [16], which has been found to impact pregnancy outcomes and gynecological diseases [17,18].

Despite the rapid growth of gynecologic cancer microbiome studies in recent years, most of them are limited to the analysis of female cancer patients with the same cancer type and the same region, the consistency of results is not strong. In addition, gynecological cancers are often difficult to detect at an early stage [19,20], and precise and scientific means of early screening are still lacking. In this study, we collected data (*n* = 529) from multiple projects of different gynecologic cancers, analyzed the vaginal microbiome of women with cancer in a multidimensional manner.

## 2 Method

### 2.1 Public data collection

We collected data from projects published on PubMed.gov containing 16S rRNA-seq for all gynecologic types of cancer (cervical, ovarian, endometrial, squamous-cell, uterine, etc.) as well as healthy populations. Ten projects with accessible sample metadata and high-throughput sequencing are included in this study. Raw sequencing data for these projects were downloaded from the National Library of Medicine and the European Nucleotide Archive via the SRA tool using the identifiers: PRJNA836143 by Mayo Clinic, PRJNA662091 [21] by Jacobson et al., PRJNA481576 [22] by Walsh et al., PRJNA758386 [23] by Gressel et al., PRJNA295859 [16] by Walther-António et al., PRJNA448161 [24] by Tsementzi et al., PRJNA725946 [25] by Fan et al., PRJNA518153 [8] by Ilhan et al., PRJNA415526 [26] by Chen et al., and PRJNA687644 [27] by Li et al.

### 2.2 Data pre-processing

VSERACH (v2.18.0) was utilized to filter the 16S rRNA data from ten projects for data merging, barcode and primer removal, quality control, etc., and the ten cohorts of clean data were merged again, and then the feature table and representative sequences were obtained through redundancy and noise reduction. Paired sequences with 97% homology to operational classification units (OTUs) were selected using the upsee-out algorithm in VSERACH to select OTUs with a mean relative abundance greater than 1 in 10,000. Using QIIME, species were labeled using a pre-trained plain Bayesian classifier by searching the Greengenes (http://greengenes.secondgenome.com/) database using default parameters (https://github.com/QIIME2/q2-feature-classifier).

### 2.3 Analysis of microbial composition and diversity

α-Diversity (Chao1, Shannon, Simpson) was assessed using the “Vegan” package (v2.5-7) running in R software v4.0.2. β-Diversity was assessed by principal component analysis (PCA) based on Bray–Curtis distance in the USEARCH (v11) platform. Statistical significance was assessed by analysis of similarity (ANOSIM). Species difference analysis was performed using the Wilcox test with a critical value of log value >2.0 and *P* < 0.01. A linear discriminant analysis (LDA) effect size (LEfSe) was used for species difference analysis at the phylum and genus level to find the biomarkers, with an LDA threshold of 2. Venn diagrams were plotted for marker comparisons.

### 2.4 Random forest model construction

We used vaginal microbial 16S rRNA-seq data to construct random forests based on genus, and the models were constructed using the “RandomForest” R package. Specifically, 70% of the data were randomly selected as the training dataset to train and construct the model, and the rest were used as the data in the validation dataset to verify the accuracy of the model. The “pROC” package was used to plot the subject operating characteristic (ROC) curve, and area under receiver operating characteristic (AUC) analysis was used to validate the fitted model.

### 2.5 Random forest model validation

To test the generalizability and robustness of the model, we performed study-to-study transfer validation and leave-one-dataset-out (LODO) validation on the entire sample based on the methods of previous researchers [28]. In the study-to-study transfer validation, we trained the classifier in a single study and evaluated its performance. Meanwhile, we applied a nested cross-validation procedure to the training studies to calculate the within-study accuracy. In LODO validation, the data from one study is set as the test set, while the data from all the remaining studies are combined as the training set.

### 2.6 Co-abundance network analysis

Correlation relationships between vaginal microorganisms in women with gynecological cancer were determined by network analysis. Taxa were represented by different node colors, node degrees by node sizes, and correlations by the thickness of the connecting lines. Associations between taxa were calculated by Spearman correlation to generate the network and visualized using Gephi (v0.9). Only connections that were significantly (*P* < 0.05) strongly correlated (≥0.7) are shown in the graph.

### 2.7 Statistical analyses

Statistical analyses were carried out in QIIME and in R packages (v4.0.2). The data of age and BMI are expressed as a mean ± standard deviation. *P* < 0.05 was considered significant.

## 3 Result

### 3.1 Characteristics of the data

In this study, we gathered ten gynecologic cancer vaginal microbiome 16S rRNA-seq data to assess vaginal microbiome differences between gynecologic cancer patients and healthy women, construct a gynecologic cancer risk prediction model, and identify biomarkers specific to the three most prevalent gynecologic cancers (cervical cancer, ovarian cancer, and endometrial cancer). We obtained a total of 529 samples from the vagina (Table 1), including the Gynecological_cancer (Gynca) group (*n* = 348) and the Control group (*n* = 181), which were subsequently grouped by cancer type, including the Cervical_cancer group (*n* = 161), the Ovarian_cancer group (*n* = 71), the Endometrial_cancer group (*n* = 101), and Normal group (*n* = 181).

**Table 1:** Details of the Bioprojects included in this study

Bioproject	Year	Location	Sample data	Age	HPV	BMI
PRJNA836143	2022	USA	OC (*n* = 26)	—	—	—
PRJNA662091	2020	USA	OC (*n* = 45)	—	—	—
PRJNA481576	2018	USA	EC (*n* = 66)	61.8 ± 10.3	—	35.2
			Normal (*n* = 5)	—		—
PRJNA758386	2021	USA	EC (*n* = 20)	—	—	—
PRJNA295859	2015	USA	EC (*n* = 15)	—	—	—
PRJNA448161	2018	USA	CC (*n* = 12)	—	—	—
			UC (*n* = 14)			
			SCC (*n* = 1)			
			Normal (*n* = 30)			
PRJNA725946	2021	China	CC (*n* = 65)	48.65 ± 6.87	HPV^−^ (*n* = 2)	24.95 ± 4.23
					HPV^+^ (*n* = 63)	
			Normal (*n* = 54)	46.81 ± 8.15	HPV^−^ (*n* = 7)	24.57 ± 3.43
					HPV^+^ (*n* = 47)	
PRJNA518153	2019	USA	CC (*n* = 10)	—	—	—
			Normal (*n* = 18)			
PRJNA415526	2017	China	CC (*n* = 9)	56.11 ± 9.02	—	23.99 ± 0.68
			Normal (*n* = 68)	43.00 ± 8.69	—	22.94 ± 2.74
PRJNA687644	2020	China	CC (*n* = 65)	—	—	—
			Normal (*n* = 6)			

### 3.2 Female menopausal status and gynecological cancer

**Figure 1:**
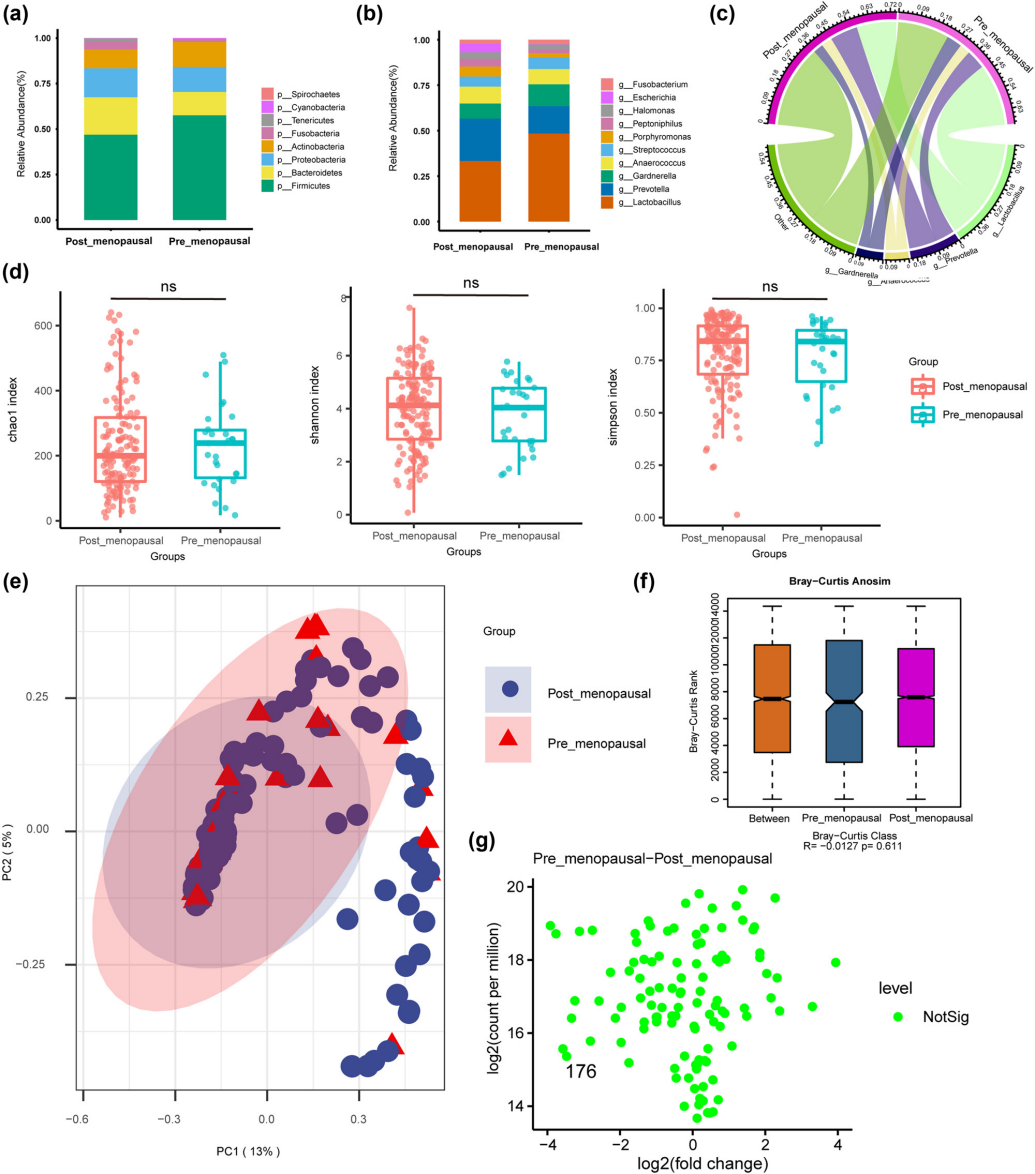
Microbial composition and difference analysis of vaginal samples in Pre_menopausal group and Post_menopausal group. (a) Stacked graph of species composition at the phylum level. (b) Stacked graph of species composition at the genus level. (c) Circle graph of species composition at the genus level. (d) Box graphs of measures of α-diversity (Chao1 index, Simpson index, and Shannon index) indices of microbial OTUs. (e) PCA based on Bray–Curtis distances for all samples from Pre_menopausal and Post_menopausal gynecological cancer patients. Ellipses represented 95% confidence level. (f) *R* and *P* values for β-diversity based on Bray-Curtis distances calculated using the ANOSIM analysis. The closer the *R* value was to 1, the greater the differences between the groups than the differences within groups; the smaller the *R* value, the less significant the differences between the groups. *P* < 0.05 showed high reliability of the test. (g) Wilcox test was used to analyze the differences between the groups. The green dots indicate that the differences are not significant.

Gynecologic malignancies are commonly observed in women who have completed menopause and perimenopause, according to previous research [29,30]. To mitigate the potential confounding influence of menopausal status on the results, we subjected gynecologic cancer samples categorized as premenopausal and postmenopausal and compared microbiome variations. We found a difference in *Firmicutes* and *Lactobacillus* abundance between postmenopausal and premenopausal women in terms of species composition (Figure 1a–c), and a decrease in *Firmicutes* and *Lactobacillus* dominance in the postmenopausal group is now a consensus trend of postmenopausal vaginal microbial alteration. Interestingly, however, the vaginal microbiome of premenopausal and postmenopausal women did not differ significantly in α-diversity and β-diversity (*P* = 0.611) (Figure 1d–f), and the Wilcox test did not show the presence of significant differences (Figure 1g).

### 3.3 Gynecologic cancer patients have unique vaginal microbiome characteristics

#### 3.3.1 Alterations in the composition of vaginal microflora associated with Gynca

**Figure 2:**
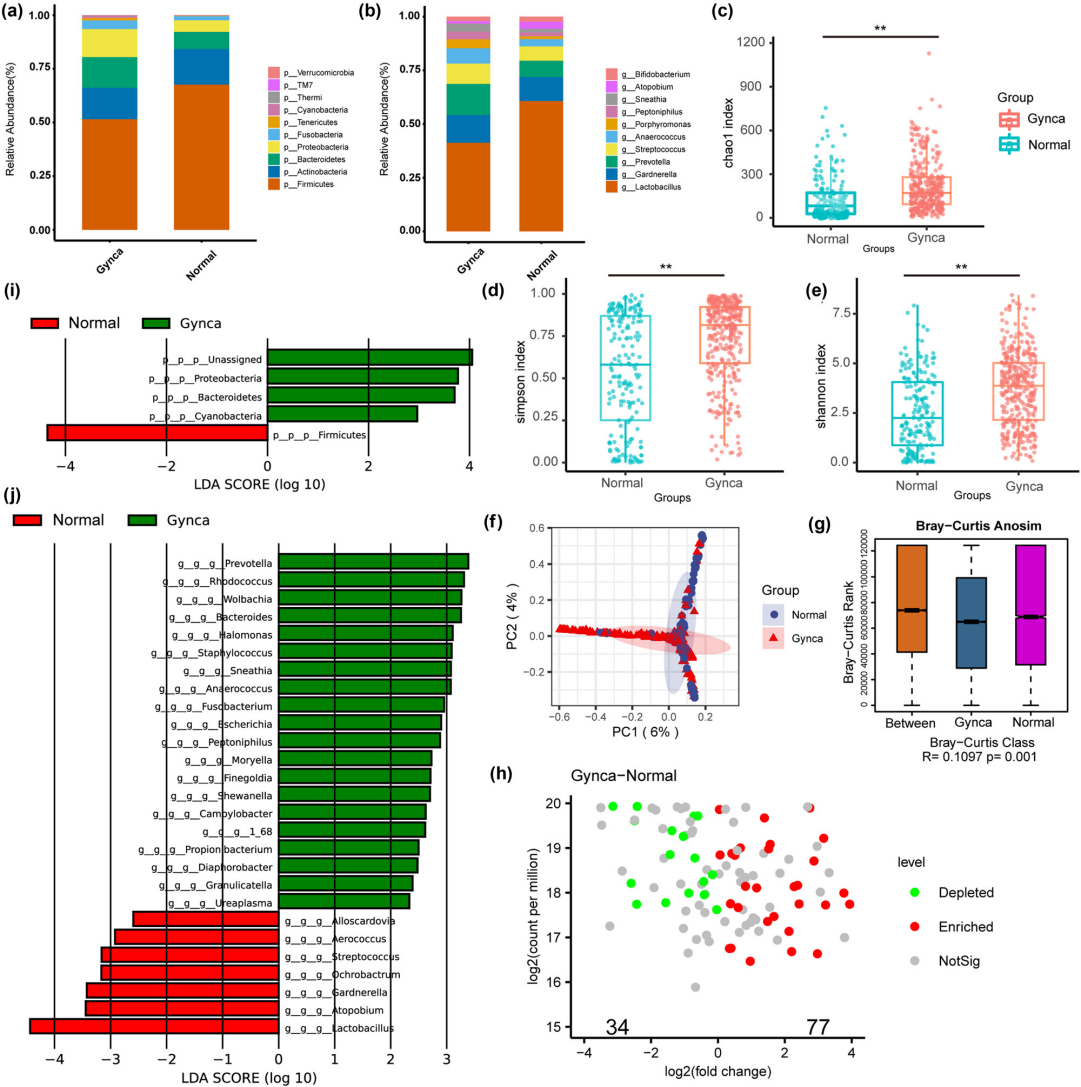
Microbial composition and difference analysis of vaginal samples in Gynca group and Control group. (a) Stacked graph of species composition at the phylum level. (b) Stacked graph of species composition at the genus level. (c–e) Box graphs of measures of α-diversity indices of microbial OTUs. (f) PCA based on Bray–Curtis distances for all samples from Gynca and Control groups. (g) *R* and *P* values for β-diversity based on Bray-Curtis distances. (h) Wilcox test was used to analyze the differences between the groups. Green and red dots indicate significantly different results, where green indicates the abundance dominance in the Control group, and red indicates the abundance dominance in Gynca group. (i) Histogram of differential enrichment phylum between Gynca and Control groups. (j) Histograms of differentially enriched genera between Gynca and Control groups. Gynca, gynecological cancer.

Following the exclusion of the influence of menopausal status on vaginal microbiology, we compiled all data to investigate the variations in the vaginal microbiome of women with gynecological cancer from different perspectives. Figure 2a and b shows the ten most abundant taxa at the phylum and genus levels, respectively. The abundance of *Lactobacillus*, *Gardnerella*, and *Prevotella* was found to be preponderant at the genus level, constituting over 50% of the overall abundance. We observed a decrease in the proportion of *Firmicutes*, *Actinobacteria*, and *Lactobacillus* and an increase in the proportion of *Bacteroidetes*, *Proteobacteria*, *Prevotella*, *Streptococcus*, and *Anaerococcus* in the Gynca group. This is a significant sign of vaginal flora dysbiosis. By comparing groups using Chao1, Shannon, Simpson vaginal microbial α-diversity indices, we demonstrated that the abundance and diversity of vaginal bacteria were substantially greater in women with gynecological cancer compared to the Control group (Figure 2c–e). By Bray-Curtis PCA, the results showed that the vaginal microbiota composition was distinguished between the Gynca and Control groups (*P* < 0.001) (Figure 2f and g). In addition, Wilcox test at the ASV level identified 111 ASVs with significantly different abundance, with 77 and 34 ASVs enriched in the Gynca and Control groups, respectively (Figure 2h). Finally, based on LEfSe analysis performed at the phylum and genus level, we found that *Proteobacteria*, *Bacteroidetes*, *Prevotella*, *Rhodococcus*, *Wolbachia*, and *Bacteroides* were enriched in the Gynca group; while in the Control group, they were enriched by *Firmicutes*, *Lactobacillus*, *Atopobium*, etc. (Figure 2i and j). Women with gynecologic cancers exhibit a more pronounced reduction in the healthy vaginal microbiome, and dysbiosis of the vaginal flora is a frequently observed characteristic.

#### 3.3.2 Vaginal microbial differences in three types of Gynca

**Figure 3:**
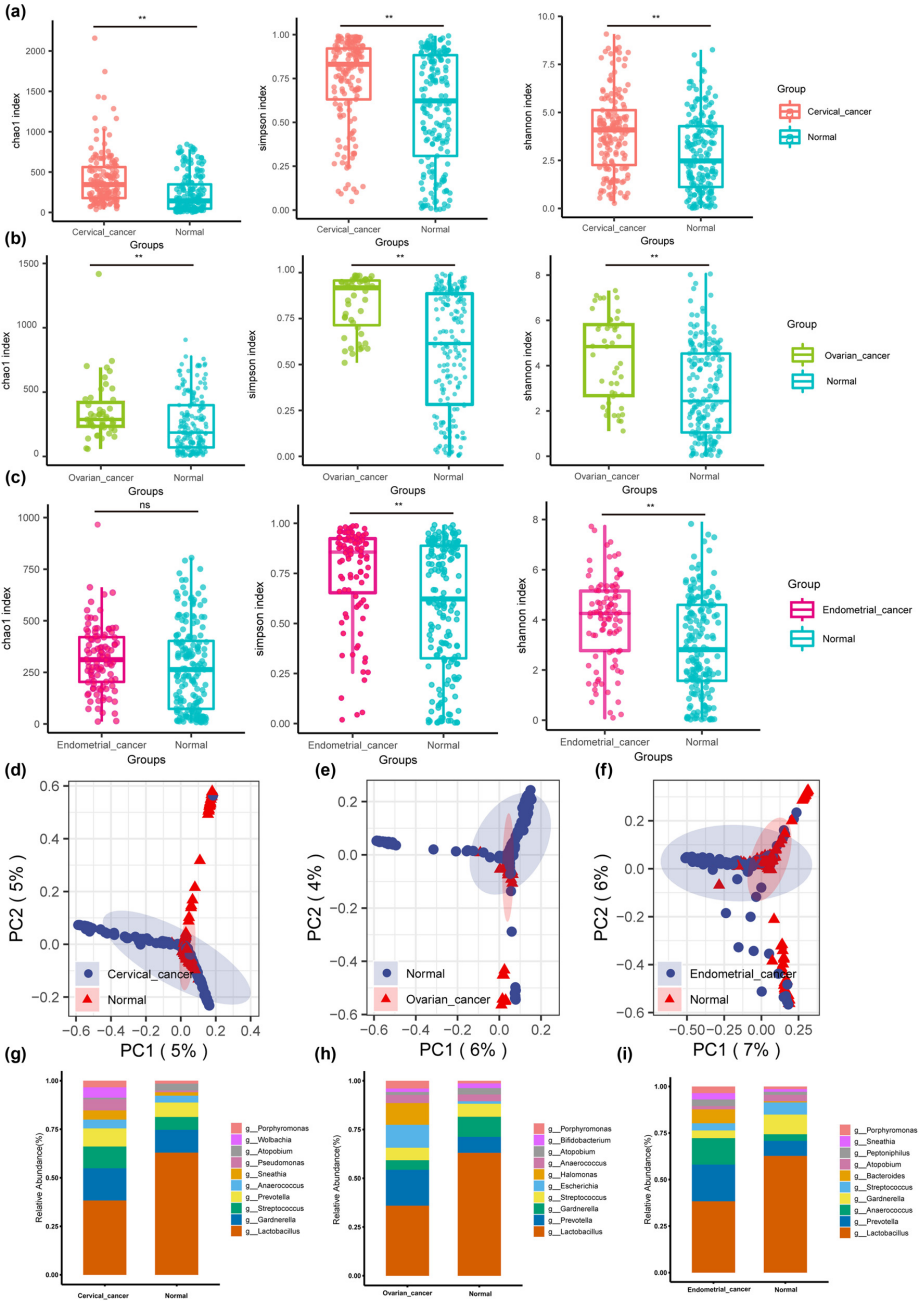
Microbial composition and differential analysis of three major gynecologic cancers (cervical, ovarian, and endometrial cancers). (a) Box graphs of the measured α-diversity indices of microbial OTUs between Cervical_cancer and Normal groups. (b) Box graphs of the measured α-diversity indices of microbial OTUs between Ovarian_cancer and Normal groups. (c) Box graphs of the measured α-diversity indices of microbial OTUs between Endometrial_cancer and Normal groups. (d) PCA based on Bray–Curtis distances for all samples from Cervical_cancer and Normal groups. (e) PCA based on Bray–Curtis distances for all samples from Ovarian_cancer and Normal groups. (f) PCA based on Bray–Curtis distances for all samples from Endometrial_cancer and Normal groups. (g) Stacked graph of species composition at the genus level between Cervical_cancer group and Normal group. (h) Stacked graph of species composition at the genus level between Ovarian_cancer group and Normal group. (i) Stacked graph of species composition at the genus level between Endometrial_cancer group and Normal group.

Additional examination of the vaginal microbiomes of cervical, ovarian, and endometrial cancer samples revealed that, in line with the overall analysis, the vaginal microbiomes of the three gynecologic cancer patients generally exhibited a greater variety and abundance, distinguishing them significantly from those of healthy samples (Figure 3a–f). Species composition tended to be disordered (depletion of *Firmicutes*, *Lactobacillus*, and enrichment of unconventional vaginal microbiome members), with depletion of *Lactobacillus* in all three gynecologic cancer groups exceeding half that of the Normal group (Figure 3g–i).

### 3.4 Mining of potential biomarkers for gynecological cancer and construction of risk prediction models

**Figure 4:**
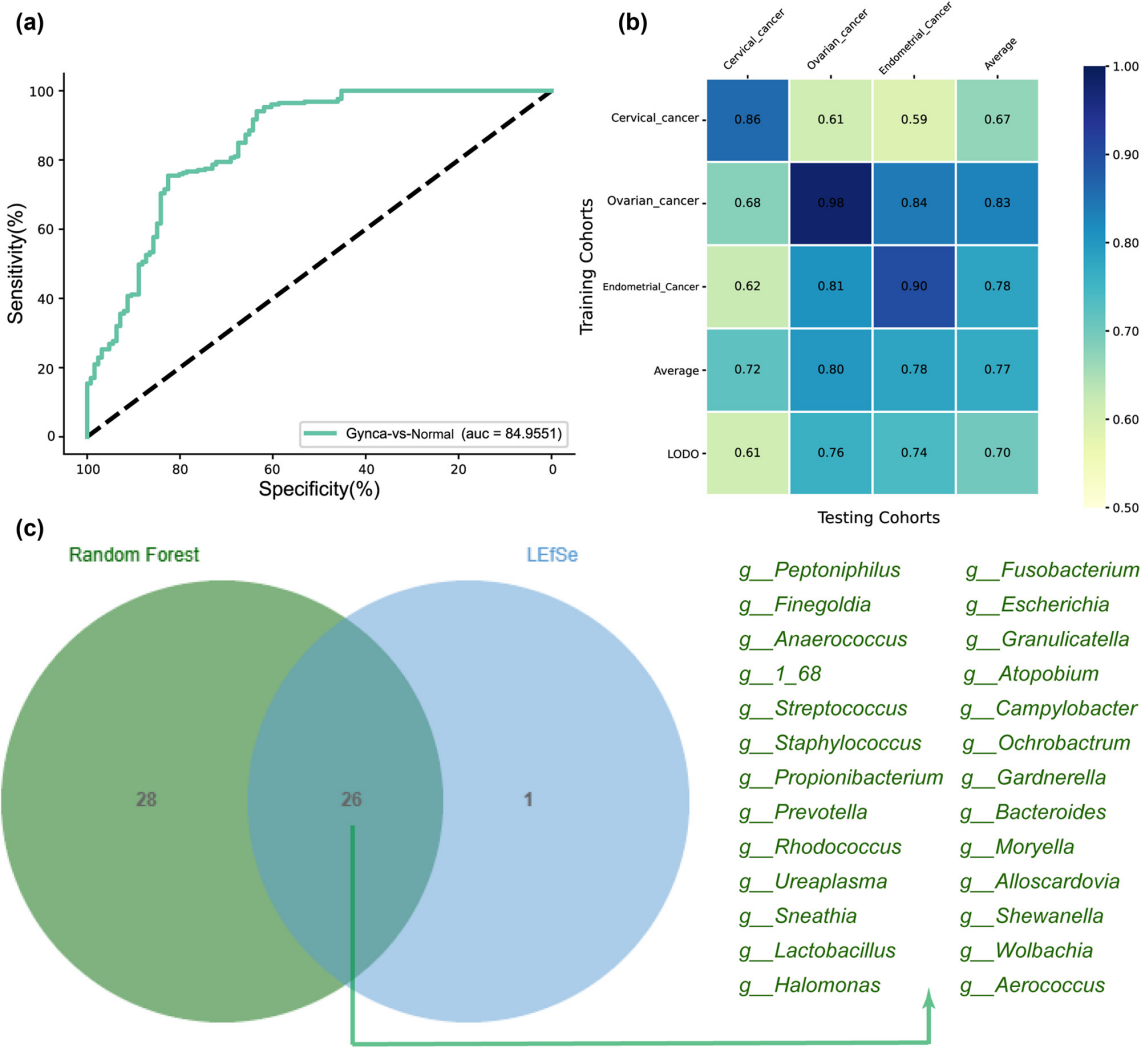
Performance of genera in early screening and diagnosis of gynecological cancer using ROC curve analysis. (a) Gynca subjects and Control group vaginal genera were tested by ROC analysis. (b) Heat map showing AUROC values for models constructed using genus characteristics in each cohort of the vaginal gynecological cancer prediction model. (c) Overlap of random forest characteristic genus with LEfSe analysis of differential genus in the Venn diagram. Gynca, Gynecological cancer.

To evaluate whether vaginal characteristic genus can be used to identify gynecological cancer patients, we constructed a 10-fold cross-validated random forest classifier. The ROC showed an AUC of 84.96% for the random forest model based on 56 characteristic genera (Figure 4a, Table 2). Figure 4c illustrates a notable rate of overlap between the characteristic genus and the differential genus derived from LEfSe analysis. This finding provides additional support for the notion that alterations in the vaginal microbiome may contribute to the development of gynecologic cancer.

**Table 2:** Microbial markers involved in random forest construction

Biomarkers	Control	Gynca	Mean decrease accuracy	Mean decrease Gini
*f_Verrucomicrobiaceae_g_Akkermansia*	8.130625837	7.981390228	10.1693016	0.349836829
*f_Veillonellaceae_g_Dialister*	1.194255489	−3.412856386	−2.000844476	0.108540437
*f_Tissierellaceae_g_WAL_1855D*	5.171008761	−0.499699073	3.932924102	0.217222638
*f_Tissierellaceae_g_Peptoniphilus*	3.014947767	1.390877945	3.75357992	0.164203009
*f_Tissierellaceae_g_Finegoldia*	−3.800142148	6.002905321	4.55298823	0.16260623
*f_Tissierellaceae_g_Anaerococcus*	10.99707312	6.095641166	12.85108446	0.682133506
*f_Tissierellaceae_g_1_68*	12.60067787	1.76911911	10.86663237	0.666871707
*f_Thermaceae_g_Thermus*	3.002369856	6.858691272	7.842029517	0.1392703
*f_Streptococcaceae_g_Streptococcus*	25.53643998	12.84395156	24.02786147	1.702967976
*f_Staphylococcaceae_g_Staphylococcus*	8.165846361	10.74503856	12.73640691	0.370328825
*f_Ruminococcaceae_g_Faecalibacterium*	8.384700377	−1.606223498	6.64500351	0.213810796
*f_Rhizobiaceae_g_Agrobacterium*	30.12847811	27.84012109	32.0959227	2.106384822
*f_Pseudomonadaceae_g_Pseudomonas*	5.457975748	7.565552243	8.677567931	0.295668966
*f_Propionibacteriaceae_g_Propionibacterium*	8.569740784	5.573278911	10.60137601	0.509739228
*f_Prevotellaceae_g_Prevotella*	−2.228729226	5.620292466	5.003047691	0.169860197
*f_Porphyromonadaceae_g_Porphyromonas*	9.323467961	4.08158266	9.467854282	0.450804465
*f_Peptostreptococcaceae_g_Peptostreptococcus*	−1.596133316	2.941351965	2.1828139	0.114553903
*f_Peptococcaceae_g_Peptococcus*	3.532271109	0.85300622	3.091388947	0.282730238
*f_Oxalobacteraceae_g_Ralstonia*	55.28656736	54.64730039	56.56342649	10.78604609
*f_Nocardiaceae_g_Rhodococcus*	11.84416411	14.89354745	17.33426406	0.878281431
*f_Mycoplasmataceae_g_Ureaplasma*	10.69172362	8.78887713	10.94887106	0.493423031
*f_Moraxellaceae_g_Acinetobacter*	5.535186843	8.424077552	8.950990882	0.329059814
*f_Leptotrichiaceae_g_Sneathia*	3.735190693	6.562793874	6.49505965	0.246821973
*f_Lactobacillaceae_g_Lactobacillus*	40.81643571	34.75834302	41.04815989	4.24709247
*f_Lachnospiraceae_g_Shuttleworthia*	11.71829449	14.85827269	14.94916199	0.610181563
*f_Halomonadaceae_g_Halomonas*	8.928191068	5.918939411	8.978388464	0.243580244
*f_Fusobacteriaceae_g_Fusobacterium*	8.302472812	3.115664493	8.618932924	0.453598561
*f_Enterobacteriaceae_g_Escherichia*	7.042481937	9.216263298	11.29157555	0.647092168
*f_Enterobacteriaceae_g_Enterobacter*	5.698888967	8.091642991	9.169832362	0.170686966
*f_Corynebacteriaceae_g_Corynebacterium*	−1.863373546	5.029912164	4.18831727	0.08922379
*f_Coriobacteriaceae_g_Unassigned*	1.372108039	4.223483687	4.26937606	0.064650275
*f_Coriobacteriaceae_g_Atopobium*	27.1097741	27.04100508	29.82148678	1.797738189
*f_Comamonadaceae_g_Pelomonas*	44.56984894	44.61587745	46.85293505	5.855587147
*f_Comamonadaceae_g_Acidovorax*	6.266470723	6.450580023	7.966480421	0.165877918
*f_Clostridiaceae_g_SMB53*	9.394722859	7.520454855	10.30003086	0.489248715
*f_Caulobacteraceae_g_Unassigned*	26.24904703	25.51608685	30.13620118	1.8349312
*f_Campylobacteraceae_g_Campylobacter*	11.65098856	4.929140317	11.62204257	0.474756115
*f_Brucellaceae_g_Ochrobactrum*	10.50976271	13.87811985	14.70803387	0.66962882
*f_Bifidobacteriaceae_g_Gardnerella*	8.785600024	8.146163905	10.87443683	0.44674851
*f_Bacteroidaceae_g_Bacteroides*	7.461755521	4.978492048	9.614458661	0.454329199
*f_Veillonellaceae_g_Megasphaera*	−1.675168596	0.22605679	−1.098843325	0.0686129
*f_Lachnospiraceae_g_Moryella*	1.123339558	1.165443468	2.195355928	0.197778515
*f_Bifidobacteriaceae_g_Alloscardovia*	12.55915455	13.55590274	14.82657561	0.490900345
*f_Actinomycetaceae_g_Actinomyces*	3.708156379	−4.987815709	−1.35495428	0.114286338
*f_Shewanellaceae_g_Shewanella*	8.952987617	7.082627587	8.993314118	0.153189286
*f_Propionibacteriaceae_g_Propionimicrobium*	4.010861158	−0.783109657	3.093319864	0.040338255
*f_Rickettsiaceae_g_Wolbachia*	6.315602866	5.806839567	7.358207092	0.182713441
*f_Actinomycetaceae_g_Mobiluncus*	5.185292613	0.76711354	4.444137075	0.107299151
*f_Enterococcaceae_g_Enterococcus*	0.855180178	3.922466182	3.771787352	0.153734201
*f_Aerococcaceae_g_Aerococcus*	−0.149417976	−0.58248759	−0.480383343	0.053894346
*f_Clostridiaceae_g_Clostridium*	1.121653282	0.624245003	1.06339699	0.08292002
*f_Carnobacteriaceae_g_Granulicatella*	0.957878156	2.326026794	2.685557543	0.044026949
*f_Bifidobacteriaceae_g_Bifidobacterium*	−2.926525324	−2.111394511	−3.246347418	0.055627807
*f_Desulfovibrionaceae_g_Bilophila*	6.631996477	3.748598764	6.296820874	0.181776495
*f_Comamonadaceae_g_Delftia*	9.253710827	4.388970065	8.636540815	0.219687561
*f_S24_7_g_Unassigned*	−1.536439334	1.104572592	−0.02063218	0.036908881

We performed study-to-study transfer validation and LODO validation to test model robustness. In the gynecologic cancer model, the AUC for study-to-study metastasis validation ranged from 0.59 to 0.98, with a mean of 0.77. The AUC for the LODO analysis ranged from 0.61 to 0.76 (mean AUC = 0.70) (Figure 4b).

### 3.5 Differences in the vaginal microbiome of women with cervical, ovarian, and endometrial cancers

**Figure 5:**
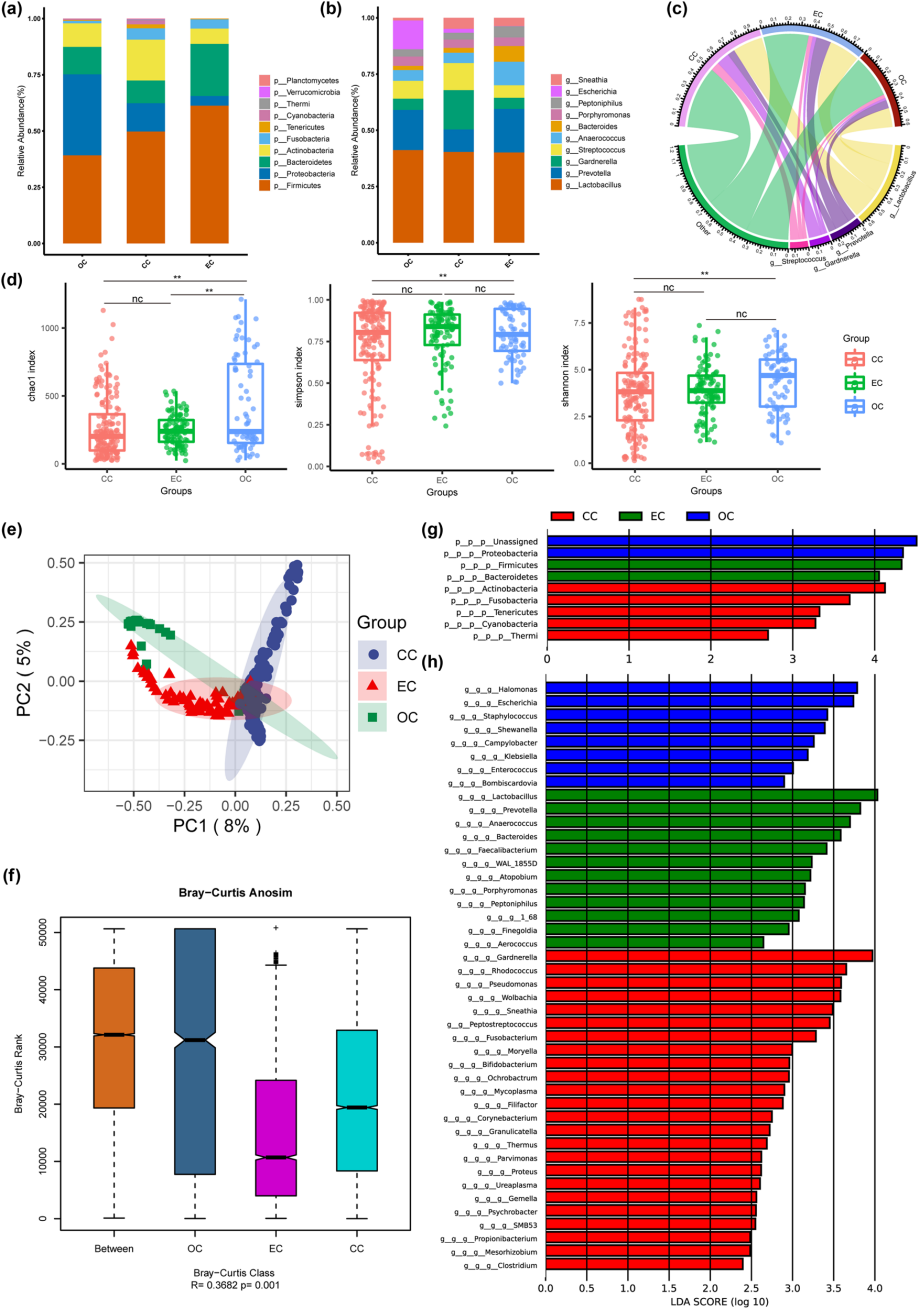
Analysis of vaginal microbial composition and differential between cervical, ovarian, and endometrial cancers. (a) Stacked graph of species composition at the phylum level. (b) Stacked graph of species composition at the genus level. (c) Circle graph of species composition at the genus level. (d) Box graphs of measures of α-diversity indices of microbial OTUs. (e) PCA based on Bray–Curtis distances for all samples from CC, EC, and OC patients. (f) *R* and *P* values for β-diversity based on Bray-Curtis distances. (g) Histogram of differential enrichment phylum among CC, OC, and EC groups. (h) Histogram of differential enrichment genus among CC, OC, and EC groups. CC, cervical_cancer; OC, ovarian cancer; EC, endometrial cancer.

To compare the variations in vaginal microbiome among the three distinct categories of gynecological cancers, we pooled all samples and analyzed them for different cancer types. Significant variations in the composition of vaginal microorganisms among women diagnosed with cervical, ovarian, and endometrial cancer are readily apparent (Figure 5a–c). *Firmicutes* abundance decreased sequentially in endometrial, cervical, and ovarian cancers, while *Proteobacteria* increased. Notably, *Lactobacillus* abundance was essentially the same in the three groups of gynecologic cancers. All three indices of α-diversity in cervical and ovarian cancers were significantly different, while Endometrial_cancer group was not significantly different from the other groups (Figure 5d). In addition, PCA of vaginal microorganisms showed significant separation of the three groups (Figure 5e and f). Additionally, LEfSe analysis was conducted to discern the distinctive microorganisms within the groups (Figure 5g and h). We found that *Firmicutes* and *Lactobacillus* were significantly enriched in the Endometrial_cancer group; *Halomonas*, *Escherichia*, and *Staphylococcus* in the Ovarian_cancer group; and *Gardnerella*, *Rhodococcus*, and *Pseudomonas* in the Cervical_cancer group.

### 3.6 Vaginas of gynecologic cancer patients show unique microbial interactions

**Figure 6:**
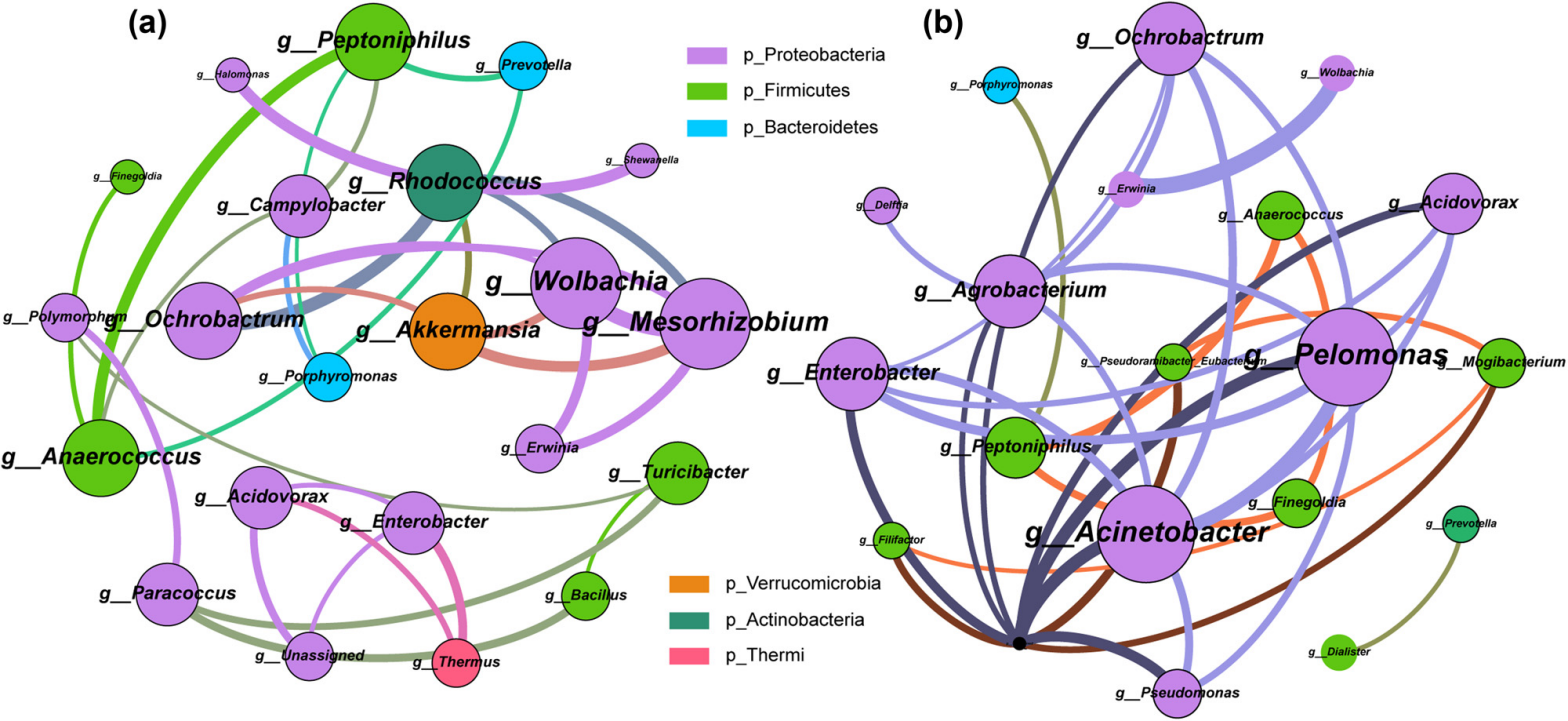
Analysis of vaginal microbial co-abundance network between Gynca and Control groups. The color of nodes indicates different phylum, node size represents node degree, connecting line indicates the interaction between genera, and the width of connecting line represents correlation. (a) Gynca group vaginal microbial co-abundance network. (b) Control group vaginal microbial co-abundance network.

To investigate the vaginal microbial interaction relationships potential alterations in gynecological cancer patients, we constructed a co-abundance network based on the species annotation outcomes of the Gynca and Control groups, respectively. We utilized Spearman correlation coefficient to evaluate the relative abundance relationships of microorganisms at the level of genus. The findings unveiled that the Gynca group exhibited a greater degree of network complexity, suggesting that vaginal microbial interactions were more robust in cases of gynecological cancer as opposed to the Control group (Figure 6a and b). In addition, the key hubs of the Gynca group were *Mesorhizobium*, *Wolbachia*, *Akkermansia*, *Ochrobactrum*, and *Peptoniphilus*, most of which are members of *Proteobacteria*, *Firmicutes* biomarkers, suggesting a change in the relevance of the differential genus to other microbial members, which may be one of the cancer causative factors.

## 4 Discussion

Gynecologic cancers pose a significant health risk to women, particularly during menopause and perimenopause. The significant association between infection and vaginal microorganisms is its primary trigger. The prevalence of cervical cancer has declined due to technological advancements that have made human papillomavirus (HPV) screening and the HPV vaccine more accessible [31]. However, there are still no efficacious preventive and therapeutic measures in place for endometrial and ovarian malignancies [32]. Large regional differences and late detection period are the main features of gynecologic cancers, and the patients with advanced cancer have a dismal prognosis. Although there is no shortage of studies related to dysbiosis affecting cancer development, most of them focus on single cancer types and specific regions. A comprehensive analysis based on a large sample size and multiple projects is necessary to explore the microbial structure of cancer risk. Therefore, we compiled vaginal microbial 16S rRNA-seq data from various gynecological cancers, including cervical, ovarian, endometrial, and other gynecological cancers. Following the exclusion of potential confounding variables based on menopausal status, a comprehensive analysis was conducted on the vaginal microbiome specific to gynecological cancer in females, as well as the patterns of flora across various forms of cancer.

In general, gynecologic cancers are more prevalent in postmenopausal and perimenopausal women, and the vaginal microbiome in postmenopausal women undergoes substantial alterations, according to previous research. Therefore, we formulated the hypothesis that the vaginal microbiome of gynecologic cancer patients might be independently influenced by menopausal status, which could compromise the validity of the current findings. Through a comparative analysis of the vaginal microbiomes of premenopausal and postmenopausal women with gynecologic cancers, we demonstrated that while minor variations in species composition existed, the overall differences were not significant. This reveals that the previous study and the subsequent analysis we performed could exclude microbiome interference from the menopausal status of the samples and the results were reliable. Variability in the outcomes of a limited number of studies may be attributable to sample and sample size variation. It is worth noting, however, that the examination of disparities during menopause was restricted to the studies incorporated in this analysis and thus was unable to ascertain a correlation between gynecologic cancer incidence and menopausal status.

Gynecologic carcinogenesis is a consequence of multiple pathological pathways. Combined with previous studies and our results, the vaginal microbiome of women with gynecological cancer is specific. A prevailing pattern is observed wherein diversity increases, *Firmicutes* decreased, *Lactobacillus* dominance was lost and pathogenic and conditionally pathogenic bacteria are enriched. The microbiome is thought to alter host immunity and influence cancer development and treatment outcomes [33]. Deviations in the composition of vaginal microbial have the potential to generate an inflammatory milieu [34] that facilitates tissue damage and contributes to disease progression and progression. Indicators of vaginal dysbiosis frequently include an enrichment of non-Lactobacillus bacteria, including conditionally pathogenic and pathogenic bacteria, and a decline in the prevalence of Lactobacillus. It is suggested that the protective effect of *Lactobacillus* on the vaginal environment, local infection, and inflammation caused by unconventional vaginal microbial enrichment may be a risk factor for gynecological cancer. *Gardnerella*, *Prevotella*, *Actinomyces*, *Porphyromonas*, *Anaerococcus*, *Peptostreptococcus*, *Streptococcus*, and *Pseudomonas* are emerging pathogens in the clinic due to their close association with pathological processes such as bacterial vaginitis, HPV infections, and precancerous lesions. These microorganisms stimulate innate immune responses in vaginal epithelial cells, resulting in the local production of cytokines and defensins. [35–41]. As an illustration, the presence and changes of group B Streptococcus, which is a member of *Streptococcus*, in the vagina can cause shedding of vaginal epithelial cells to induce rising infection and inflammation [42], stimulate host immune response [43], and is detected at a higher rate in older women [44]. Group B Streptococcus colonization is impacted by dysbiosis of the vaginal flora, altered vaginal pH, decreased abundance of *Lactobacillus*, and increased non-commensal bacteria. In addition, exposure to *G. vaginalis* encourages the colonization of the vagina by group B Streptococcus [45]. *Gardnerella* and other vaginal non-commensal bacteria in the Gynca group increased in parallel with the proportion of *Streptococcus*, whereas *Lactobacillus* abundance decreased, as demonstrated by our findings. In addition to this, pro-cancer effects of the microbiota include genotox release, inhibition of apoptosis, stimulation of proliferation, and promotion of genomic instability [46]. *Campylobacter* enriched in the Gynca group is closely related to gastrointestinal diseases [47], revealing potential migration and exchange of gut-vaginal flora. In addition, *Campylobacter jejuni* is capable of producing a genotoxin (cytolethal distending toxin) that acts as a tumorigenic agent [48]. *Fusobacterium nucleatum* affects cancer cell proliferation by promoting genomic instability and mutations. Consistent with the findings of previous research, our analysis of vaginal flora of patients with three prevalent gynecologic malignancies revealed that the distribution of pathogenic bacteria differed among the different cancer types. Significantly, pathogenic bacteria enriched in the endometrium, including *Prevotella*, *Atopobium*, *Anaerococcus*, *Porphyromonas*, and *Peptoniphilus*, were also enriched in the vagina of patients with endometrial cancer, were also enriched in the endometrium [3], revealing a potential correlation between pathogenic bacterial enrichment and carcinogenesis.

Previously, machine learning-based microbiome approaches were also employed to detect patients with gynecologic cancer-related conditions. In 2020, Kang et al. conducted an analysis of the stool microbiome associated with early invasive cervical cancer and distinguished patients based on a machine learning classifier model with an AUC of 0.913 [49]. Subsequently, in 2021, Li et al. investigated endometrial cancer using microbiome and transcriptomic techniques and revealed that microbial markers *Prevotella* and serum D-dimer, which are fibrin degradation products showed high potential (AUC = 0.86) for predicting endometrial carcinogenesis [50]. Notwithstanding the high predictive value shown by the above models, their brief sample sizes and superficial analysis constitute certain limitations. In addition, the application of machine learning model construction extended to the prediction of chemotherapy resistance and the detection of precancerous lesions. Lee and colleagues’ model for identification of cervical intraepithelial neoplasia based on the vaginal microbiome with 33 characteristic genera had an AUC of 0.952 [51]; Gong et al. showed an AUC of 0.909 [52] for an random forest model for predicting chemotherapy resistance in ovarian cancer based on gut microbes; Wang et al. distinguished neoadjuvant chemotherapy response in locally advanced cervical cancer based on *Bacteroides* with an AUC of 0.84 [53]. Random forest models constructed using machine learning based on the microbiome can contribute to the construction of predictive diagnostic models for future diseases. Besides the obvious advantages of non-invasive, inexpensive, and highly acceptable, it can also advance the time to early screening and diagnosis to a certain extent, which is of great value for diseases whose prognosis is affected by patients staging.

One notable characteristic that sets this study apart from others is its comprehensive examination of gynecological malignancies, including the dynamic vaginal microbiome. Numerous factors are known to influence the human microbiome, and even minor modifications to the human body can result in substantial alterations to the microbiome. Our sample comprised individuals hailing from different countries, ethnicities, with different lifestyles and significantly different physical conditions, and we could not exclude the interference of these factors in the results. Nevertheless, the generalizability of the acquired results is beyond dispute. Because of the complexity and diversity of the samples included, the results are representative of a wide range of people. The sample size is large enough to dilute to some extent the effect of excessively varied data and extreme data on the results. In addition, we have compared the microbiomes of three types of gynecologic cancers, making the results both more applicable and specific, enabling analysis of differences across populations and regions. Our results contribute to future development and research on non-invasive screening, decrease the financial burden of screening for a broader spectrum of women, mitigate regional disparities, enable gynecologic cancers to be identified more accurately at an earlier stage, enhance the efficacy of subsequent therapies, and contribute to the global cancer burden.

Nonetheless, this study is not devoid of limitations; the precision of 16S rRNA-seq analysis at the species level is low, and we were unable to acquire outcomes pertaining to vaginal microbial species, while the metabolism and effect of distinct species may have large differences, constituting the source of error in our findings. Furthermore, the data we pooled were characterized by too few controls, and the introduction of external controls could potentially introduce additional errors.

In light of current results and trends, it is imperative that women of reproductive age undergo routine vaginal examinations. Early detection and intervention for gynecologic cancer among women will be one of the most effective cancer control strategies of the future. Cancer triggers are diverse and the microbiome is equally influenced by multiple factors. Further research should follow up with patients and prospectively capture multiple indicators of their age, ethnicity, disease history, sexual life, and diet. Avoid errors in results due to non-cancerous factors. How to organically combine microbiome screening with other tests to achieve non-invasive, accurate, and efficient, should also be the focus of future research.

## 5 Conclusion

In summary, we demonstrated that women with gynecologic cancer have a unique vaginal microbiome. The dysbiotic vaginal flora of gynecologic cancer patients is generally characterized by increased diversity and abundance, loss of dominance of *Lactobacillus*, and an increase in conditionally pathogenic bacteria. These variants can predict unique interactions between the host and certain bacteria or metabolites, which may help to explore the pathogenesis of gynecological cancers in the future.
